# 3D Microstructure Effects in Ni-YSZ Anodes: Influence of TPB Lengths on the Electrochemical Performance

**DOI:** 10.3390/ma8105370

**Published:** 2015-10-21

**Authors:** Omar M. Pecho, Andreas Mai, Beat Münch, Thomas Hocker, Robert J. Flatt, Lorenz Holzer

**Affiliations:** 1 Institute of Computational Physics, Zurich University of Applied Sciences, Winterthur 8400, Switzerland; hoto@zhaw.ch (T.H.); holz@zhaw.ch (L.H.); 2 Institute for Building Materials, ETH Zurich, Zurich 8093, Switzerland; flattr@ethz.ch; 3 Hexis SA, Winterthur 8404, Switzerland; andreas.mai@hexis.com; 4 Empa, Materials Science and Technology, Laboratory for Concrete and Construction Chemistry, Dübendorf 8400, Switzerland; Beat.Muench@empa.ch

**Keywords:** cermet, degradation, microstructure, tomography, polarization resistance, solid oxide fuel cells, Ni-YSZ, redox cycling, triple phase boundary (TPB)

## Abstract

3D microstructure-performance relationships in Ni-YSZ anodes for electrolyte-supported cells are investigated in terms of the correlation between the triple phase boundary (TPB) length and polarization resistance (*R*_pol_). Three different Ni-YSZ anodes of varying microstructure are subjected to eight reduction-oxidation (redox) cycles at 950 °C. In general the TPB lengths correlate with anode performance. However, the quantitative results also show that there is no simplistic relationship between TPB and *R*_pol_. The degradation mechanism strongly depends on the initial microstructure. Finer microstructures exhibit lower degradation rates of TPB and *R*_pol_. In fine microstructures, TPB loss is found to be due to Ni coarsening, while in coarse microstructures reduction of active TPB results mainly from loss of YSZ percolation. The latter is attributed to weak bottlenecks associated with lower sintering activity of the coarse YSZ. The coarse anode suffers from complete loss of YSZ connectivity and associated drop of TPB_active_ by 93%. Surprisingly, this severe microstructure degradation did not lead to electrochemical failure. Mechanistic scenarios are discussed for different anode microstructures. These scenarios are based on a model for coupled charge transfer and transport, which allows using TPB and effective properties as input. The mechanistic scenarios describe the microstructure influence on current distributions, which explains the observed complex relationship between TPB lengths and anode performances. The observed loss of YSZ percolation in the coarse anode is not detrimental because the electrochemical activity is concentrated in a narrow active layer. The anode performance can be predicted reliably if the volume-averaged properties (TPB_active_, effective ionic conductivity) are corrected for the so-called short-range effect, which is particularly important in cases with a narrow active layer.

## 1. Introduction

**Figure 1:**
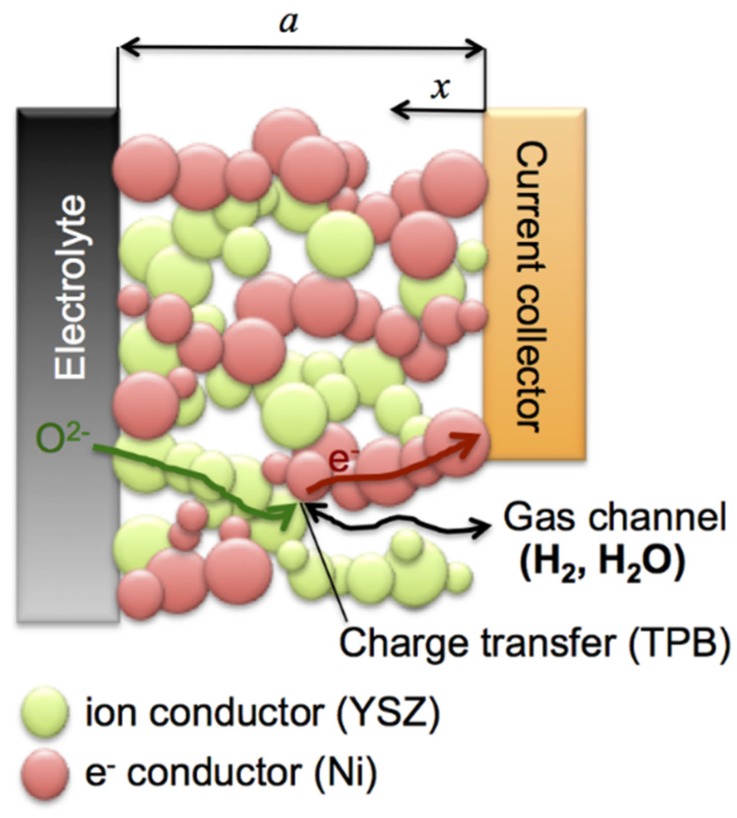
Schematic representation of Ni-YSZ cermet anode and the mechanism of charge transport and charge transfer processes.

Porous Ni-YSZ is a state-of-the-art anode material for SOFC applications. The anode reaction mechanism includes a coupling of ionic and electronic charge transport with charge transfer, as illustrated in Figure 1. Each phase is responsible for a specific transport process, *i.e.*, oxide ions in YSZ, electrons in Ni, and gas/fuel in the pores. The charge transfer reactions take place at the triple phase boundary (TPB). The microstructure of the fresh anode material has a strong impact on the initial performance as well as on the long-term stability and lifetime.

The aim of this study is to unravel the complex relationships among microstructure, anode performance, and degradation behavior upon redox cycling, with a strong focus on the influence from variations in TPB density. The complexity of this topic is mainly due to the fact that the anode performance results from coupled transfer and transport processes. Thereby the charge transfer and the associated ionic and electronic current densities may spread over the anode in different ways, depending on the microstructure and materials properties. For example, if TPB and associated charge transfer kinetics are rate limiting then the electrochemical reaction tends to spread over a relatively thick active layer. In contrast, if the ionic conductivity is relatively low, then the active zone tends to be restricted in a narrow active layer close to the electrolyte-anode interface. Microstructure degradation usually leads to a decrease of both ionic transport as well as charge transfer properties. It is thus not easy to predict how microstructure degradation affects the distribution of current densities and electrochemical activity. The property, which degrades less strongly, usually has a dominating influence on current distribution and in this way it buffers the more severe decrease of the other property and its influence on performance. Hence, knowledge about the distribution of current densities and electrochemical activity is a key to a profound understanding of the relationship between microstructure and anode performance.

Numerical modeling can be used to simulate coupled transport and transfer reactions in order to obtain profiles of current densities throughout the anode [1,2,3]. However, in order to capture the influence of microstructure in a reliable way, the model requires input from detailed 3D-analysis. In our approach, the 3D investigations of fine-, medium-, and coarse-grained anodes are split into two separate publications. A previous paper was focusing on the relationship between topological parameters and effective transport properties [4]. The present work is dealing with total and active triple phase boundary (TPB) and their relation with anode performance (*R*_pol_).

**Figure 2:**
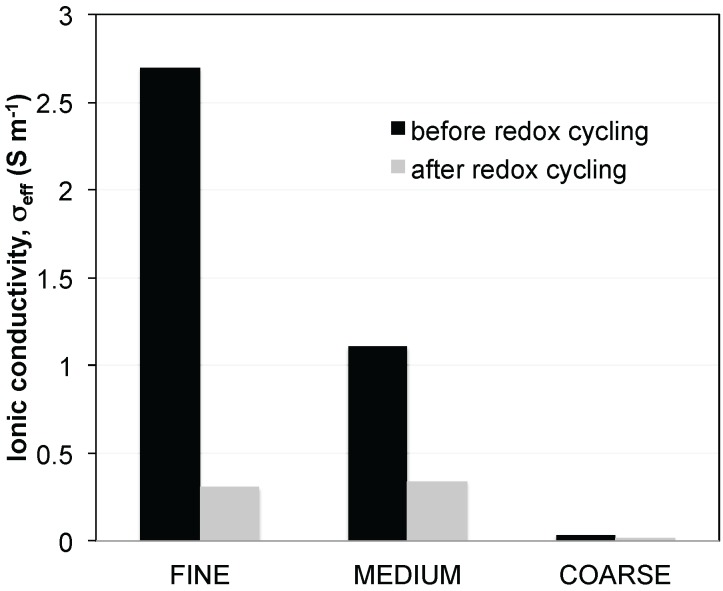
Ionic conductivities in Ni-YSZ anodes as a function of microstructure coarseness. Conductivities (σ_eff_) are predicted based on an empirical relationship using transport relevant topological parameters (σ_eff_ = σ_0_ Φ*_eff_* β^0.36^/τ^5.17^) [5,6]. These parameters are obtained from 3D-analysis. σ_0_ = intrinsic ionic conductivity of YSZ (σ_0,YSZ@950 °C_ = 15.6 S·m^−1^), Φ*_eff_* = effective volume fraction, β = constrictivity, τ = tortuosity.

Since *R*_pol_ is dependent on both TPB and transport properties, we briefly summarize the findings of the previous publication [4]. It was shown that the effective ionic and electronic conductivities are related to three main topological factors (*i.e.*, effective volume fraction Φ*_eff_*, constrictivity β and tortuosity τ) of YSZ and Ni, respectively. Thereby, it was also observed that degradation of fine and coarse anodes have very different characteristics:

The fine anode initially has a dense microstructure due to its relatively high sintering activity of the fine YSZ grains. The two main degradation features upon redox cycling are irreversible volume expansion and Ni agglomeration. The ionic conductivity decreases in the fine anode, which is mainly due to a reduction of bottleneck dimensions in YSZ (*i.e.*, decrease of constrictivity β). The ionic conductivities of fine, medium, and coarse anodes are shown in Figure 2. For the coarse anode the main degradation feature upon redox cycling is the loss of YSZ connectivity, which is attributed to weak bottlenecks associated with the low sintering activity of coarse YSZ grains. As shown in Figure 2, the ionic conductivity is already very low in the initial state due to the low constrictivity (β). Redox cycling affects all three parameters (Φ*_eff_*, β, and τ) of the coarse YSZ phase so that the ionic conductivity drops to almost 0 (S/m). In contrast, the electrical conductivity of the coarse anode does not degrade significantly upon redox cycling.

The present paper now focuses mainly on the relationship between TPB and anode performance, thereby also taking into account the main findings on effective conductivity. In this context it should be noted that the investigated cells are electrolyte-supported (ESC). Because of the small anode layer thickness (20 μm) used in ESC in combination with the high intrinsic conductivity of Ni (σ_0,Ni@950 °C_ = 2.21 × 10^6^ S·m^−1^), limitations from electrical conductivity can usually be neglected. Hence in our cells the performance is mainly controlled by charge transfer kinetics (including TPB) and effective ionic conductivity.

In literature, the TPB length is often directly correlated with the electrochemical performance [7,8]. A simplistic view of this correlation is an inverse relationship between the polarization resistance and the TPB length. This kind of correlation was observed for first generation anodes, which consist only of a single electronically conductive phase (e.g., Pt). For example, works of Virkar *et al.* on single-phase cathodes (*i.e.*, patterned Pt [9] and Sr-doped LaMnO_3_ (LSM) [10]) show that the area-specific resistance (*R*_pol_, polarization resistance) is inversely proportional to TPB length. The linear relationship between 1/*R*_pol_ and TPB is often also attributed to composite electrodes. However, in composite anodes, ionic and electronic transport resistances usually also contribute to *R*_pol_. The anode polarization thus contains two main contributions (in button cells) that are tightly coupled to each other:

*R*_pol_ = *R*_trans_ + *R*_act_ (1)

where *R*_trans_ is the resistance from transport of electrons and oxide ions in the anode and *R*_act_ is the resistance related to the electrochemical charge transfer at the TPB. For anodes with a large contribution from *R*_trans_, the simplistic relationship between 1/*R*_pol_ and TPB is no longer true.

In experimental studies using impedance spectroscopy the different contributions from transport and transfer are usually not easy to distinguish (see supplementary material, Figure S1). However, it is well-understood that symmetrical semicircular curves indicate the absence of transport limitations (*i.e.*, *R*_pol_ = *R*_act_), whereas asymmetrical such as “teardrop” shapes may be the result of mixed limitations from transport and activation, or complete domination by transport resistances [11,12].

In our work we use a finite element model (DC, macrohomogeneous) for coupled charge transport and transfer [2], which provides the distribution of current densities and electrochemical transfer rates throughout the anode layer. Based on these distribution profiles the two contributions (*R*_trans_ + *R*_act_) contained in the polarization resistance can be described quantitatively. In the present paper we use the model to describe some basic scenarios that correspond with typical microstructure characteristics of the investigated anodes.

**Figure 3:**
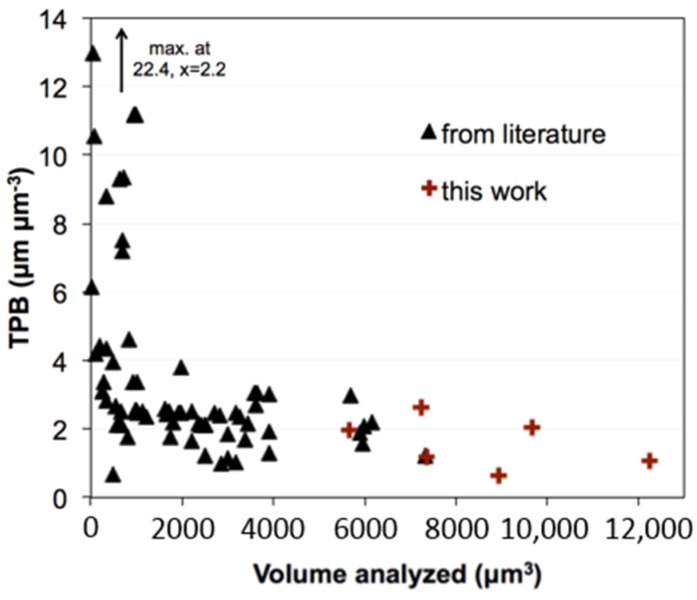
TPB lengths in Ni-YSZ anodes as a function of volume analyzed from literature [7,8,13,14,15,16,17,18,19,20,21,22,23,24,25,26,27,28,29,30,31,32,33,34,35,36,37] (data table with references are given in the supplementary material, Table S1). Independent from the fabrication method, the TPB lengths increase significantly for some of the analyses that are taken on a very small volume (which is probably due to limitations of representativeness).

In literature [7,8,13,14,15,16,17,18,19,20,21,22,23,24,25,26,27,28,29,30,31,32,33,34,35,36,37] some very high TPB values (up to 22.4 μm^−2^) are reported for Ni-YSZ anodes. As shown in Figure 3, those high TPB values are exclusively measured for small sample volumes (<1000 μm^3^). Therefore, the high TPB values are most probably an artifact from lack of representative elementary volume (REV). In fact, all data points from larger sample volumes (>1000 μm^3^) exhibit TPB values in a relatively narrow range between 1 and 4 μm^−2^. More explanation about compilation of Figure 3 is given in the supplementary material.

In this study, we are particularly interested in the quantitative correlation between the electrochemical performance and TPB lengths. Therefore, active and total TPB lengths need to be distinguished from each other. The active TPB lengths are obtained considering the connectivity in each of the three phases (*i.e.*, pores, Ni, and YSZ). The influence of redox cycling on the electrochemical performance and on the microstructure is established in order to unravel the degradation mechanisms. Furthermore, limitations to the transport of mobile species are discussed for three different microstructure scenarios. Thereby it is shown that the microstructure limitations strongly influence the distribution of electrochemical activity and also of ionic and electronic current densities within the anode. Overall, for composite Ni-YSZ anodes the relationships between electrochemical performance and microstructure, which is not a simple function of the active TPB length can be rationalized through the complex interplay of charge transport and transfer processes, and the mechanistic scenarios proposed in this work.

## 2. Experimental

### 2.1. Anode Fabrication, Redox Cycling, and Impedance Measurements

A detailed description of the anode fabrication, exposure to redox cycling and electrochemical impedance measurements can be found in the work of Iwanschitz *et al.* [38,39]. As a summary, three different anodes with fine, medium, and coarse microstructures are produced by screen printing, oxidized sintering and subsequent reduction. To vary the microstructure three different powders (fine, medium, coarse) of 8YSZ (Mel Chemicals, purity >99%) are mixed always with the same powder of NiO (J.T. Baker, purity >99%) to form Terpineol-based slurries. The ratio of the powders is chosen such that the solid volume fraction (Ni:YSZ) after reduction is 40%:60%. The Terpineol-based slurries are then screen-printed onto 3YSZ substrates (Nippon Shokubai, 140 um) to form button-cells with porous LSM/YSZ as cathode. The anode layer thicknesses of the fine, medium, and coarse are 20, 25, and 26 μm, respectively. The anodes are sintered in air at 1350 °C for 4 h after screen-printing. Subsequent reduction is performed at 950 °C. The redox experiments were conducted at 950 °C in combination with impedance-measurements. Eight redox cycles were carried out manually. The anode was oxidized due to the back-diffusion of oxygen from the post-combustion zone, which was located in close proximity to the anode when the H_2_ gas flow was shut down abruptly for 30 min. The flow of the non-humidified cathode air was not interrupted during the experiment. The reduction of the anode was carried out by ramping-up the H_2_ flow within 5 min. The cells were characterized electrochemically without polarizing the cells during the experiments except during the current/potential measurements. The impedance curves were recorded at the beginning and after each cycle using a Zahner IM6 impedance analyzer in the frequency range between 20 mHz to 200 kHz with a 20 mV amplitude.

### 2.2. Image Acquisition by FIB-Tomography and SEM

**Figure 4:**
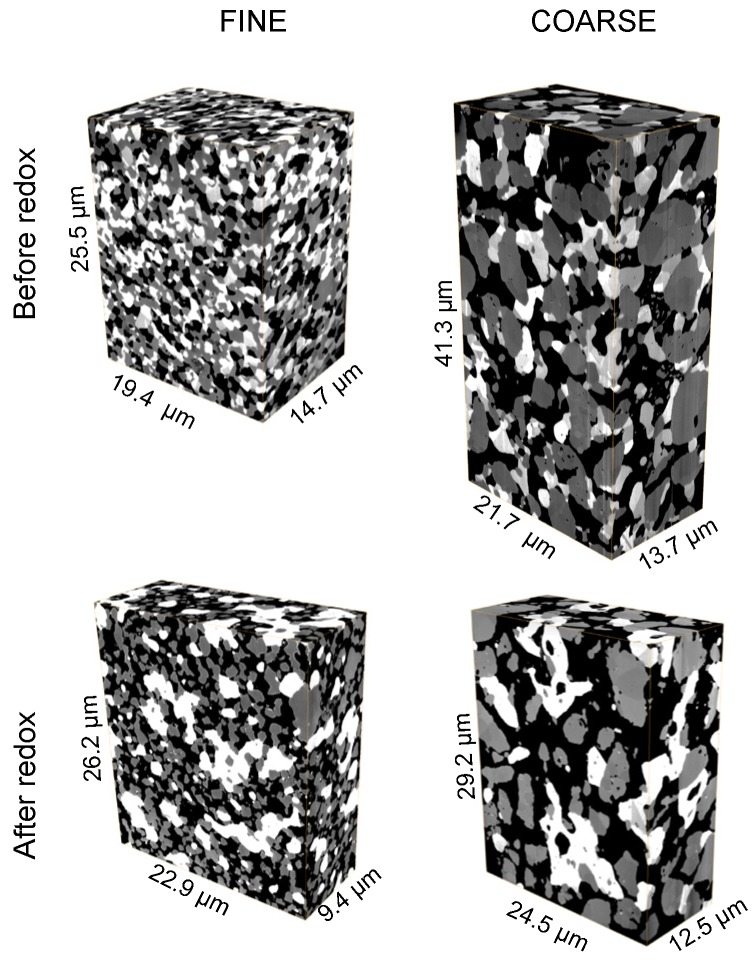
3D reconstructed microstructures of fine and coarse anodes before and after redox cycling obtained from FIB-tomography. Pores (black), YSZ (gray), and Ni (white). Significant Ni coarsening is visible in the samples after redox cycling. Note: Additional visualizations of all three anodes (fine, medium, coarse) are given in previous publications [4,40].

For microstructure analysis, polished cross-sections were prepared from the samples that were used for 4-point conductivity measurements. The porous anodes were first impregnated with low-viscosity resin, cut through, and then polished on textile substrates with 6, 3, and 1 μm diamond suspensions. Focused ion beam (FIB)-SEM tomography was performed with a Helios Nanolab 600i (FEI), hosted at ETH Zürich (ScopeM). FIB tomography includes a repetitive procedure of alternating ion sectioning and SEM imaging. The serial sectioning was done with an ion beam current of 2.5 nA and an accelerating voltage of 20 kV. For serial SEM imaging good contrast settings for Ni-YSZ was achieved with the so-called through-the-lens detector (TLD) at 2.0 kV accelerating voltage and 0.69 nA beam current. The 3D reconstructed images of fine and coarse Ni-YSZ anodes before and after redox cycling are shown in Figure 4.

### 2.3. Determination of Triple Phase Boundary Length (TPB)

3D image stacks obtained by FIB-tomography were pre-processed before the analysis of TPB. The pre-processing protocol includes image alignment, selection of the region of interest (ROI), noise filtration, and segmentation of the phases (*i.e.*, pore, Ni, and YSZ). Fiji [41], including some in-house developed plug-ins and Avizo [42] software packages were used to execute the pre-processing protocol. Details on image processing can be found in our previous study [4,40]. The corresponding dimensions of the tomographic data (Number of voxels in x, y, z/Voxel size/Cube dimensions) are summarized in the supplementary materials of Ref. [4].

The TPB analysis includes the following steps: After segmentation, each of the phases is indexed uniquely. For each voxel, the index values of the adjacent voxels are determined and examined. Thereby, if two different values are detected, the voxel is identified as a border voxel located at an interface. If there are three different values, the voxel is identified to be located at a triple phase boundary (TPB). Subsequently, a binary mask including the TPB voxels is created. Following the above evaluation scheme, the following parameters can be determined: (1) Total number of pixels assigned to any TPB object; (2) Total number of discrete TPB objects; (3) Total number of two phase interfaces, and of pixels at each interface boundary; (4) The length of each TPB line is determined based on the skeletonization of TPB-voxels in each object; (5) Furthermore, active TPBs are distinguished from non-active TPBs using a connectivity check. At each TPB, the percolation of each phase towards each direction (*i.e.*, to six directions in 3D) can be determined as the presence of an unbroken connection from the phase to the border. This feature is calculated by running region-growing processes from the examined borders. In active TPBs the connectivity check in each phase has to be positive for one specific direction (e.g., Ni has to be connected with the current collector side). An example output of the TPB analysis with connectivity checks, including a correction procedure for boundary truncation effects, is shown in the supplementary material (Table S2). Truncation effects at cube boundaries may introduce apparent interruptions of the percolating pathways. These truncation effects are suppressed by analyzing TPBs within a central sub-cube of reduced size, whereas the corresponding connectivity checks are performed within the total cube volume.

## 3. Results

**Figure 5:**
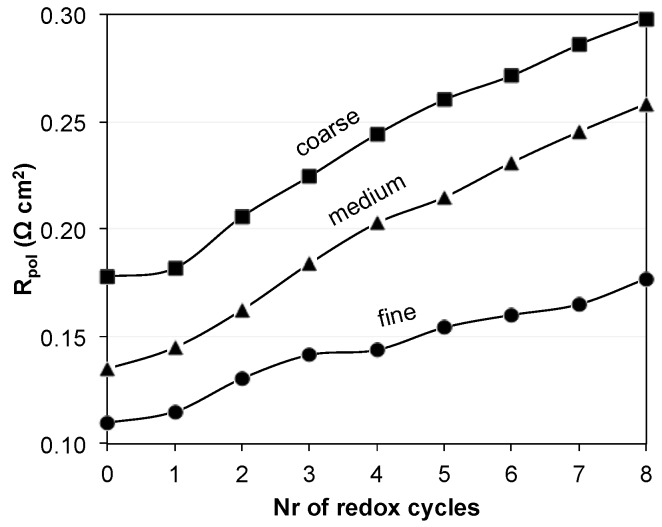
Evolution of polarization resistance (*R*_pol_) of Ni-YSZ anodes during exposure to eight redox cycles at 950 °C (adapted from Iwanschitz [38]). Degradation is stronger in medium and coarse samples compared to the fine anode.

Figure 5 shows the evolution of the polarization resistance (*R*_pol_) of the fine, medium, and coarse anodes upon redox cycling. The degradation of *R*_pol_ over eight redox cycles is stronger in coarse and medium samples compared to the fine one, which is indicated by steeper slopes for medium and coarse anodes in Figure 5. The initial *R*_pol_ (0th) increases from fine to coarse. The change in *R*_pol_, given by the difference between the 0^th^ and 8^th^ cycles (initial and final resistances) is similar for the medium and coarse anodes (*ca.* 0.120 Ω·cm^2^), while it is smaller for the fine anode (0.07 Ω·cm^2^).

**Figure 6:**
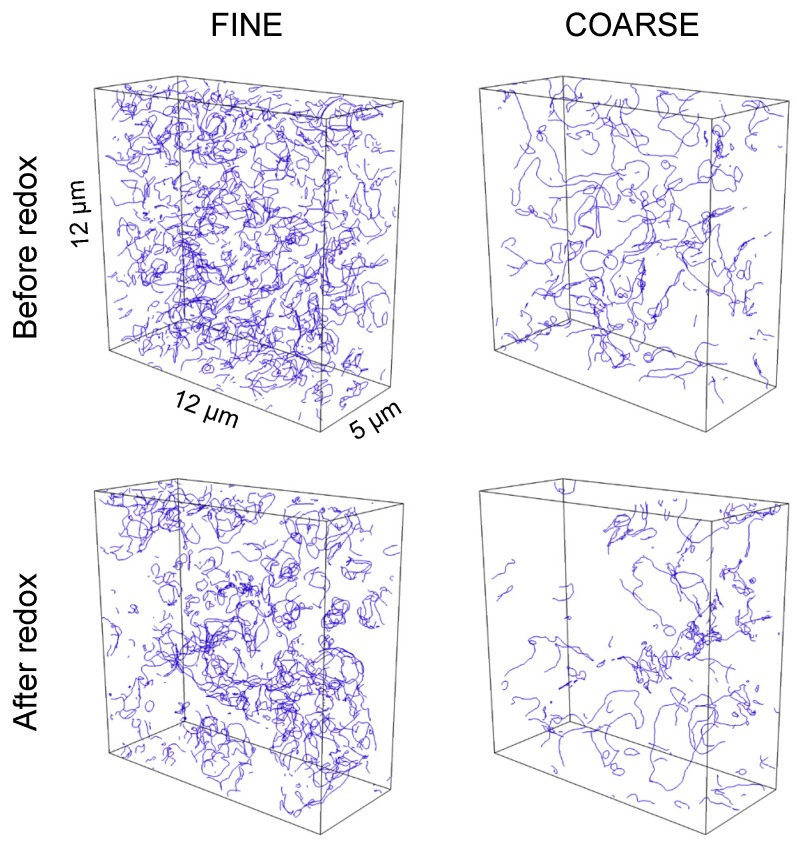
TPB lines (blue) determined from tomographic data for the fine and coarse anodes. Redox cycling obviously leads to a decrease of TPB density. Note: Some of the TPBs are not active, due to loss of phase connectivity in one of the transporting phases (not shown). Loss of connectivity is particularly strong for the YSZ phase in the coarse anode after redox cycling (bottom right). Total and active TPB lengths are given in Figure 7.

Figure 6 shows the TPB lines obtained from image analysis of Ni-YSZ tomographic data. There is a clear dependency of TPB density on the fineness of the initial microstructure. Moreover, Figure 6 also illustrates a reduction of TPB density due to redox cycling. It must be emphasized that these TPB lines are only active for electrochemical reactions if all three phases merging at the TPB are connected to their base. For example YSZ has to be connected with the electrolyte (located on the left side of the cubes in Figure 6) in order to enable transport of oxygen ions from the electrolyte to the TPB. Similarly the pores must be connected with the gas channel and Ni with the current collector (both on the right side of the cubes in Figure 6).

**Figure 7:**
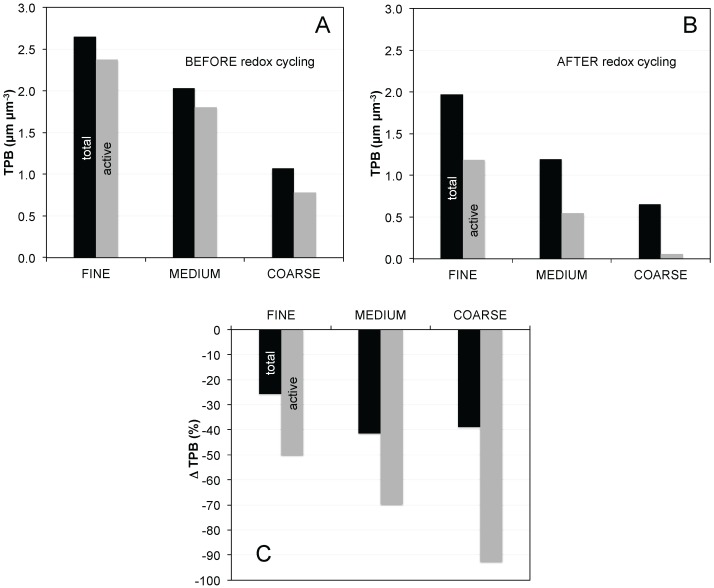
Densities of total and active triple phase boundary lengths (TPB_total_, TPB_active_) for the different Ni-YSZ anodes before (**A**); and after (**B**) redox cycling; (**C**) change in TPB_total_ and TPB_active_ before and after redox cycling (relative to before redox cycling).

Figure 7A,B show the TPB_total_ and the corresponding TPB_active_ for the different anodes before and after eight redox cycles. In the initial microstructures (Figure 7A), about 90% of TPB_total_ in fine and medium is active, while it is lower for the coarse anode (73%). After redox cycling (Figure 7B), about 60% of the TPBs are still active in the fine anode, and 46% in the medium anode. However, only 9% remain active in the coarse anode. Hence, the effects from loss of connectivity upon redox cycling are much stronger in the coarse anode.

Upon redox cycling, the decrease of TPB_total_ in the medium and coarse samples are 41% and 39%, respectively, whereas for the fine anode the relative change is only 26% (Figure 7C). The drop of TPB_active_ is significantly higher than the one measured for TPB_total_. TPB_active_ decreases by *ca.* 50% in the fine anode, 70% in medium, and 93% in the coarse anode.

The changes of TPB lengths presented in Figure 7 are related to some characteristic features of redox degradation, which are strongly dependent on the initial anode microstructure. Some of these characteristics (including volume fractions, connectivity, bottleneck dimensions, constrictivity, and tortuosity) have been presented in a previous publication (see Ref. [4]). Here we briefly summarize the most important characteristics of the initial microstructures and of the associated redox degradation. Thereby we define the fine and the coarse anodes as “end-members” with different degradation mechanisms.

**Figure 8:**
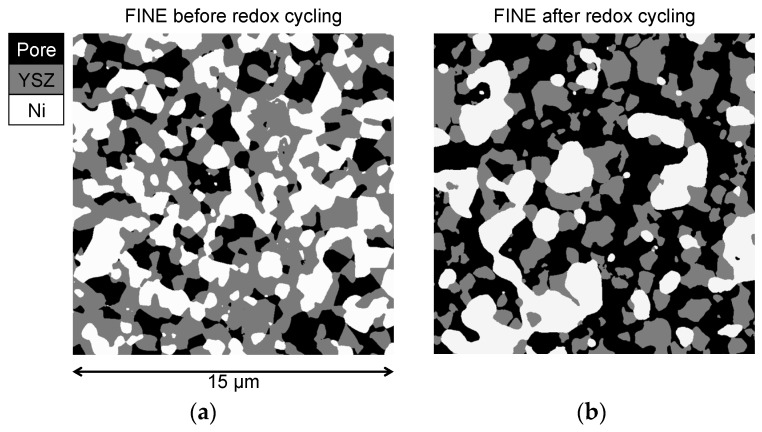
Microstructures of the fine anode before (**a**); and after redox cycling (**b**). After redox cycling, Ni forms larger particles and some isolated islands, which results in a loss of TPB_total_ and TPB_active_.

The microstructure characteristics for the fine anode are illustrated in Figure 8. It shows 2D segmented images for the fine anode before and after redox cycling. The initial microstructure of the fine anode is relatively dense and has a porosity of 25 vol %. In the pristine sample the Ni phase (white) is homogeneously distributed. Repetitive redox cycling leads to irreversible volume expansion of the anode layer, so that the porosity increases to 47 vol %. After redox degradation Ni is concentrated in larger patches and also some isolated Ni particles exist. The coarsening and inhomogeneous redistribution of Ni results in a decrease of TPB_total_. Nevertheless, a significant portion of the Ni phase is still connected (18% of Ni) and forms a percolating network. Therefore the loss of TPB_active_ in the fine anode is moderate (*i.e.*, 40%).

**Figure 9:**
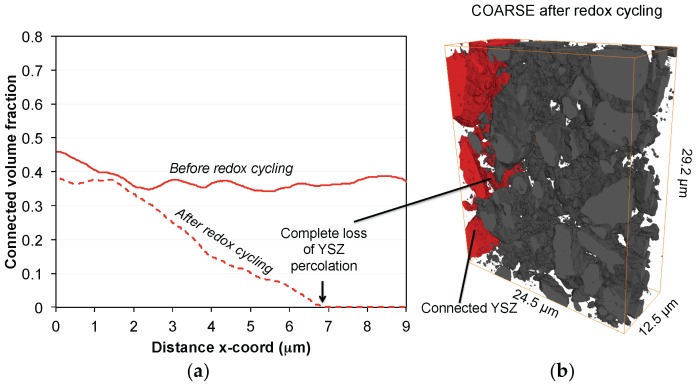
(**a**) Profiles of connected volume fractions of YSZ in the coarse anode as a function of the distance to the electrolyte-anode interface (x-direction). After redox cycling, complete loss of percolation is observed at *ca.* 7 μm; (**b**) 3D reconstructed image of YSZ showing the connected particles (red) confined to a region near the electrolyte-anode interface.

In contrast to the fine anode, the initial microstructure of the coarse anode is characterized by a relatively high porosity of 39 vol %. The lower density compared to the fine anode is due to a lower sintering activity of the large YSZ particles. This low sintering activity also leads to relatively narrow bottlenecks and to a low constrictivity (β) for the coarse YSZ (β_coarse_ = 0.01 β_fine_ = 0.37). The difference in the initial microstructure (coarse *vs.* fine) also leads to a different degradation behavior. First of all, Ni agglomeration is less severe in the coarse. This is illustrated by lower loss of percolation and also by lower decrease of electrical conductivity (In fact, the electrical conductivity in the coarse anode even increases during the first eight redox cycles). The second important difference is the fact that connectivity of YSZ in the coarse anode is drastically reduced, which is visualized in Figure 9. At a distance of *ca.* 7 μm from the anode-electrolyte interface, the connected volume fraction of YSZ drops to 0. This loss of connectivity is attributed to strain localization at weak YSZ bottlenecks. Hence, although the Ni degradation is less severe in the coarse compared to the fine anode, the influence of Ni-NiO transformation on the YSZ-connectivity appears to be much more detrimental in the coarse anode. Due to the breakdown of the YSZ-connectivity also the TPB_active_ is reduced by 93%. Assuming a direct correlation between TPB_active_ and R_pol_, significant performance degradation (>>100%) must be expected in the coarse anode. However, as shown in Figure 5, the anode still exhibits an acceptable performance (increase of *R*_pol_ by approximately 60%). These findings indicate that the complete loss of TPB_active_ does not necessarily lead to an anode failure. Possible explanations for such controversial observations shall be discussed below.

## 4. Discussion

**Figure 10:**
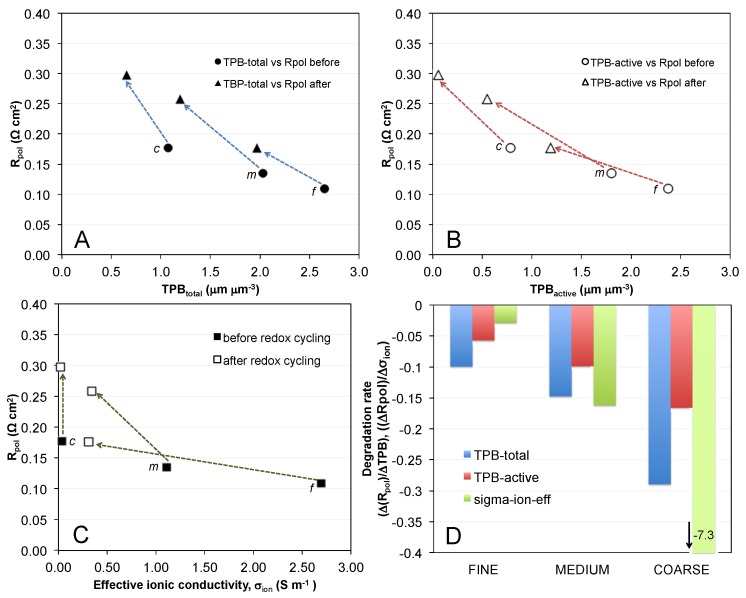
Polarization resistance (*R*_pol_) *versus* (**A**) TPB_total_; and (**B**) TPB_active_ before and after redox cycling. Degradation ratios (indicated by arrows) include a y-component for the changes in polarization resistance (*R*_pol_) and an x-component for changes of TPB. The slopes of the degradation are shown as a bar plot (**D**). It documents that the influence of ΔTPB on Δ*R*_pol_ does not follow a constant slope. The degradation ratios Δ*R*_pol_/ΔTPB increase from fine to coarse; (**C**) The relationship between *R*_pol_ and effective ionic conductivity shows a similar pattern of degradation ratios with steeper slopes for coarser microstructures.

The results can be summarized by three main points: (1) The initial microstructure and the associated TPB lengths inversely correlate with *R*_pol_. Finer microstructures with higher TPB exhibit lower *R*_pol_, as shown in Figure 10; (2) Such a correlation also exists for the samples that were exposed to redox cycling, in the sense that the decrease in anode performance scales with the decrease in TPB length. Thereby, the coarser the initial microstructures the higher are the degradation rates of both TPB and *R*_pol_; (3) However, TPB and anode polarization (*R*_pol_) are linked in a complex way, which cannot be directly expressed in a simple, linear relationship. The third point contradicts the frequent interpretation that *R*_pol_ can be easily related to TPB length. This simplistic interpretation neglects the interplay of charge transfer reactions with the various charge transport processes (see Figure 1).

Figure 10 represents a comparison of polarization resistances with (A) TPB_total_ length; (B) TPB_active_ length; and (C) the effective ionic conductivity (YSZ). (Note: Effective ionic conductivities are taken from a previous paper dealing with transport properties in the same Ni-YSZ anodes. See Ref. [4]). We define the so-called degradation ratio (Δ*R*_pol_/Δx), where x is either TPB_total_, TPB_active_ or the effective ionic conductivity. This ratio (shown in Figure 10D) represents the sensitivity of *R*_pol_ with respect to changes of TPB and conductivity, respectively. The decrease in TPB (both total and active) upon redox cycling is accompanied by the increase in *R*_pol_ as shown in the degradation ratios (Figure 10A,B, blue and red arrows). However this ratio is different for fine, medium, and coarse samples. It is worth noting that the degradation ratios for ionic conductivity (Figure 10C) show a similar behavior as for TPB, with shallow slopes for the fine and steeper slopes for the coarser sample. Despite the strong degradation of ionic conductivity and TPB_active_ in the fine anode, the degradation of *R*_pol_ is not very severe in this sample. In contrast, medium and coarse anodes show a strong increase of *R*_pol_.

Overall, Figure 10 documents that *R*_pol_ and TPB do not follow a simple linear relationship, otherwise the data points in Figure 10A,B, would plot on straight lines and the degradation ratios would be equal for fine, medium, and coarse. The changing values of the degradation ratios indicate that there is no simple and linear micro-macro-relationship (Δ*R*_pol_/ΔTPB), neither for TPB_total_ nor for TPB_active_.

The data points for the coarse anode in Figure 7 require particular attention. As mentioned earlier the TPB_active_ drops to almost 0 upon redox cycling (see Figure 7B). In addition, the effective ionic conductivity is very low already in the pristine state due to narrow bottlenecks and it drops to 0 upon redox cycling due to loss of percolation. The question arises, why low TPB_active_ combined with a breakdown of phase network for ionic conduction does not lead to a complete failure of the anode.

In order to answer this question, one would have to consider the distribution of ionic and electronic current densities throughout the anode, together with the associated electrochemical transfer activity. In electrochemical impedance measurements, the electrode contributions of transport processes and electrochemical reactions to the overall polarization usually cannot be easily distinguished from each other and are often reported as a single value (*R*_pol_). This corresponds to the width of the corresponding arc in the Nyquist plot. From numerical simulation, however, it is well understood that the anode resistance (*R*_pol_) results from a complex interplay between charge transport (ions, electrons, gas) as well as from charge transfer. Rüger *et al.* [1] for example has presented current density profiles through the anode for different microstructures. These profiles nicely document how current densities and associated electrochemical activities are distributed depending on the effective conductivities and on the TPB densities.

Since these coupled phenomena are hardly distinguishable by EIS measurements, we consider mechanistic scenarios with different limiting processes in order to explain the observed non-linear relationships between *R*_pol_ and TPB. For this purpose we use a macro-homogeneous 1D finite-element model (FEM) to simulate the anode reaction mechanism. Ionic and electronic transport processes are described by Ohm’s law and charge transfer at the TPB is described with the Butler-Volmer equation (see Refs. [2,3]). This model takes into account microstructure characteristics of the anode due to the input of effective ionic and electric conductivities, as well as TPB from separate microstructure analyses. A detailed description of the model as well as a detailed discussion of simulation results will be presented in a follow-up paper. Here we summarize, in a simplistic way, some simulation results for basic scenarios that help to explain the observed non-ideal relationship between TPB and R_pol_ in Ni-YSZ anodes.

We distinguish the following three anode scenarios that are discussed on a qualitative level: (1) no transport limitation of mobile species, neither ionic nor electric; (2) limitation of ionic transport (*i.e.*, low ionic conductivity); and (3) extreme limitation of ion transport and reduction of TPB_active_ due to complete loss of YSZ connectivity. Scenario 1 represents an idealized anode. Scenario 2 is comparable with the degradation phenomena observed in the fine anode, whereas scenario 3 corresponds with the degradation phenomena observed in the coarse anode.

**Figure 11:**
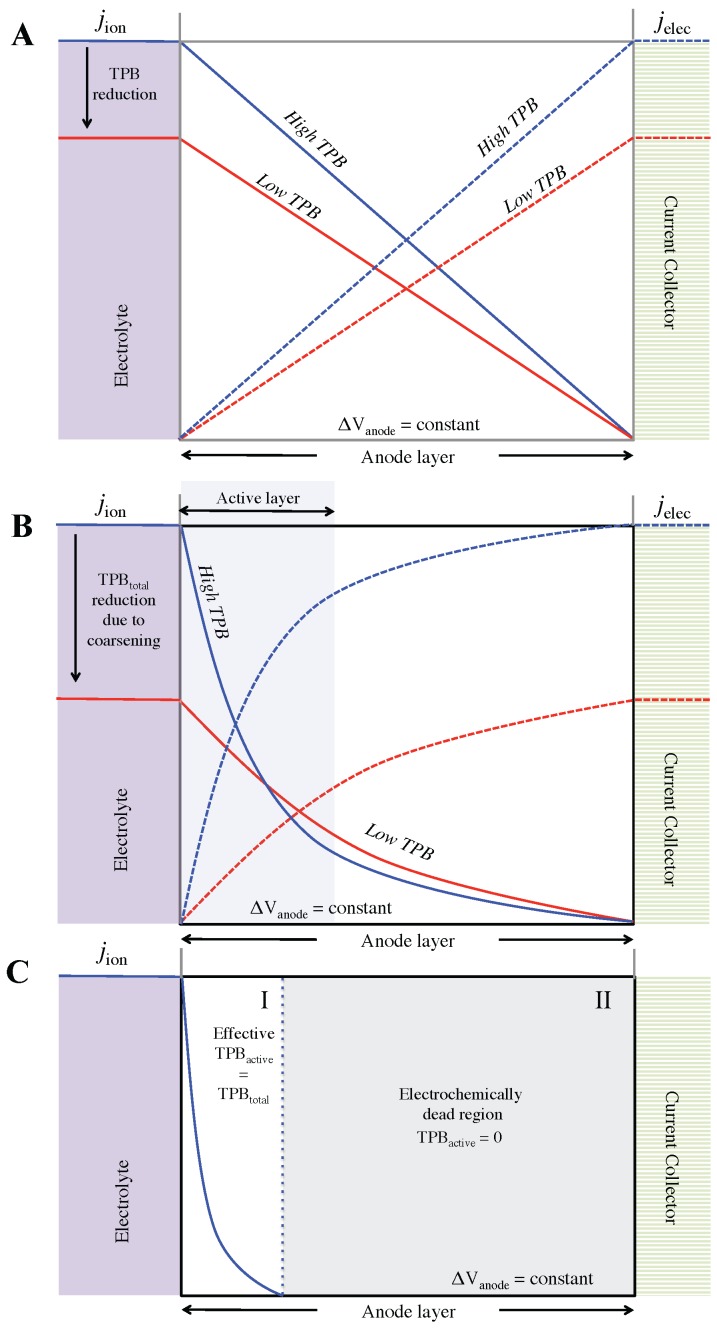
Schematic representation of current density distributions for three different anode scenarios, where j_ion_ decreases and j_elec_ increases from left to right (j_tot_ = constant): (**A**) Idealized scenario where no limitation on transport of mobile species exists. The whole anode acts as an active layer with constant electrochemical activity; (**B**) Anode scenario with limitations for ionic transport. This leads to a concentration of charge transfer reactions in the active layer and associated inhomogeneous distribution of current densities. This scenario is representative for most Ni-YSZ anodes, including Ni-coarsening as observed in the fine anode; (**C**) Anode scenario with low ionic conductivity including a complete loss of percolation for YSZ in domain II. This scenario is representative for the coarse anode before and after redox cycling. (*j* = current density, Δ*V* = voltage drop).

For all scenarios we apply a constant voltage drop over the anode and consider the corresponding current (*j*) profiles depending on TPB lengths and ionic conductivity. In any case, the total charge current density is constant: *j*_total,x_ = *j*_ion,x_ + *j*_elec,x_. The current distribution profiles for the first case are shown in Figure 11A. For scenario 1 there is no transport limitation. Therefore the entire anode is being used for the charge-transfer reaction. The slope (Δ*j_ion_*/Δ*x*) is constant, which signifies constant electrochemical activity throughout the entire anode. The decrease of TPB length (as indicated with red color in Figure 11), still assuming no transport limitation, leads to changes in total current density (*j*_total,x_) and electrochemical activity, as indicated by a shallower slope (Δ*j*/Δ*x*). It must however be emphasized that this simplified scenario is not realistic. Indeed, in our anodes relatively strong transport limitations arise from the rather low intrinsic ionic conductivity of the ceramic phase (σ_0,YSZ@950 °C_ = 15.6 S·m^−1^). These limitations are enhanced by microstructure effects, which lead to effective ionic conductivities in the range 0.02–2.7 S·m^−1^, as documented in a previous study [4]. In contrast, intrinsic electric conductivity of Ni is much higher (σ_0,Ni@950 °C_ = 2.21 × 10^6^ S·m^−1^), so that electric transport limitations can usually be neglected for thin anodes in electrolyte-supported cells.

The second scenario is more realistic since it takes into account the influence of ionic transport resistance. As shown in Figure 11B, the system optimizes the distribution of transport and transfer reactions in order to minimize energy losses. In this second scenario (compared to the first one without transport limitations), the transport distances of ions are reduced and the electrochemical activity is concentrated within a limited zone called the active layer. The slope (Δ*j*/Δ*x*) also becomes steeper as the active layer moves closer to the electrolyte-anode interface and deviates from linearity, in contrast to the simplified scenario in Figure 11A. Consequently, the charge-transfer activity is highest near the electrolyte-anode interface. Similar current density profiles are reported e.g., in the modeling work of Rüger *et al.* [1].

Upon degradation the TPB_active_ decreases, which leads to a drop of the total current density (at constant cell voltage). Such a scenario corresponds with the case “fine anode degradation” in Figure 7 and Figure 8. The TBP degradation also leads to a shallower slope (Δ*j*/Δ*x*), as illustrated by the red curve in Figure 11B. This implies lower electrochemical activity and as a result, the active layer is again spread (change in thickness of active layer) to compensate for the loss of TPB. Hence, qualitatively two opposing effects are identified from degradation: Ionic transport limitations have the tendency to decrease the active layer thickness (shorter transport distances), whereas loss of TPB leads to an increase of active layer thickness (increase of absolute TPB-number). Usually the redox degradation affects both transport properties as well as TPB-lengths. Thereby, the thickness-change of the active layer upon degradation cannot easily be predicted. This thickness and its change upon degradation can only be determined with a suitable model describing coupled transport and charge transfer processes, which also takes into account the relevant microstructure characteristics and effective properties of the anode.

Figure 11C shows the current distribution profiles for an anode where loss of YSZ connectivity is present. This setting is observed in the coarse anode after redox cycling, which is also illustrated in Figure 9. At some distance away from the electrolyte-anode interface the loss of YSZ leads to a drop of TPB_active_ to zero. These distant regions are electrochemically dead (domain II in Figure 11C). However, close to the electrolyte-anode interface (domain I in Figure 11C) where ionic transport distances are short, the loss of percolation (at longer transport distances) has no detrimental effect so that the electrochemical activity is linked mainly with TPB_total_ (*i.e.*, effective TPB_active_ at short distances ≈TPB_total_). This phenomenon is called “short-range effect”. It means that, close to the interface the influence of microstructure cannot be explained by volume averaged parameters such as TPB, constrictivity (β), tortuosity (τ), and effective conductivity (σ*_eff_*_,*io*_). When transport distances are shorter than the size of the representative elementary volume (REV), then volume averaged microstructure characteristics and associated effective properties are not relevant for the reaction kinetics. Hence in the active layer close to the interface the microstructure limitations are overestimated if they are described by volume averaged parameters (TPB, β, τ, σ*_eff_*). Some important microstructure limitations, which are due to tortuous pathways, narrow bottlenecks and loss of connectivity (and active TPB), require transport distances similar as the REV-dimensions, in order to become relevant. Consequently, in order to capture microstructure effects reliably in a macro-homogeneous model, one has to take into account this so-called short-range effect. This is of particular importance for electrode scenarios with a narrow active layer.

The postulation of a short-range effect provides an explanation for the complex pattern in the measurements as discussed earlier for the coarse Ni-YSZ anode after redox cycling. As shown in Figure 7, TPB_active_ in the coarse anode decreases from 0.8 to 0.06 μm^−2^ (*i.e.*, by 93%). Hence, with a simplistic view on TPB-performance relationships it would be expected that this anode should exhibit a very low electrochemical performance. Nevertheless, although a significant increase of *R*_pol_ is measured (from 0.18 to 0.30 Ω·cm^2^), this increase is much lower than expected based on the measured changes in TPB_active_. The short-range effect thus provides an explanation, in the sense that Δ*R*_pol_ does not correlate with ΔTPB_active_, because loss of percolation in YSZ is only relevant at longer transport distances.

In contrast to TPB, *R*_pol_ is not a volume average property but it is the result of coupled transport and transfer processes that lead to uneven distributions of current densities throughout the anode. In particular, as shown in the third scenario, limitations due to loss of percolation are compensated by the concentration of the electrochemical activity in a narrow active layer close to the electrolyte, where (almost) all TPBs are active due to short transport distances for oxygen ions. Consequently it is assumed that the effective TPB activity changes across the anode layer—from the short distances, where all TPBs are active, to longer distances, where electrochemical activity correlates with TPB_active_. The more limiting the TPB_active_ at long distances, the stronger the concentration of electrochemical activity close to the electrolyte-anode interface will be. But a complete loss of TPB_active_ (as a volume averaged microstructure characteristic) does not necessarily lead to anode failure, nor does a loss of effective ionic conductivity.

## 5. Conclusions

In literature, the electrochemical performance (*i.e.*, *R*_pol_) of Ni-YSZ anodes is often directly linked with the length of active TPBs. The variation of *R*_pol_ for different microstructures upon redox cycling clearly shows the influence of the TPB density (*i.e.*, inverse relationship). However, contrary to the simplistic view of a linear relationship between *R*_pol_ and TPB, the results of this work suggest a more complex correlation between these two parameters in composite anodes. *R*_pol_ is influenced also by charge transport, and especially the contributions from ionic transport limitations on anode performance may be a reason for the observed deviation from linearity.

We identify two main scenarios that are suitable for a qualitative description of microstructure-performance relationship upon degradation of Ni-YSZ anodes. “Scenario 2” represents the degradation behavior of the fine and medium grained anodes. They are dominated by Ni coarsening, which leads to a reduction of TPB lengths. In addition, the microstructure degradation also leads to a drop of ionic conductivity due to changes in tortuosity and constrictivity (bottleneck effect). Both degradation phenomena (reductions of TPB_active_ and ionic conductivity) may have opposite effects on the distribution of current densities and active layer thicknesses.

In scenario 3, which is compatible with degradation phenomena observed in the coarse anode, the loss of YSZ percolation leads to a complete loss of active TPB and breakdown of effective ionic conductivity in the anode layer. However, the anode remains electrochemically active because charge transfer gets localized near the anode-electrolyte interface. This case also illustrates some limitations of conventional approaches used to establish quantitative microstructure-property relationships. Hereby the macroscopic electrode behavior is dominated by a process, which takes place in a thin active layer close to the interface. It is important to note that the properties of the thin active layer are not equivalent with the effective properties of the bulk material. This is described by the so-called short-range effect.

As an outlook, the microstructure parameters related to the charge-transport (Φ, β, τ) and charge-transfer (TPB) processes can be used as an input for numerical simulations of the anode reaction mechanism. In order to reliably predict the distribution of current densities and electrochemical activity as well as to quantify the different contributions from transfer and transport on the polarization resistance, it is necessary to consider the short-range effect. Such insights obtained from the quantification of microstructure effects in combination with modeling do not only give an in-depth understanding and a more differentiated interpretation of composite anode degradation, but may also be useful in the conceptualization of new anode designs and microstructure optimization.

## Supplementary Materials

The following are available online at www.mdpi.com/1996-1944/8/10/5370/s1.
