# Supplementary material for: 3D Microstructure Effects in Ni-YSZ Anodes: Influence of TPB Lengths on the Electrochemical Performance
Source: Materials (Basel). 2015 Oct 21;8(10):7129–44. doi: 10.3390/ma8105370 (PMC5455394; doi:10.3390/ma8105370)
Supplement: Supplementary file 1 [file materials-08-05370-s001.pdf]

Supplementary Materials

Table S1. Summary of TPB lengths determined by several groups available in literature.

Authors	Pub. Year	Method	TPB (µm µm^{-3})		Volume under study (µm^3)
			total	active	
Wilson [13]	2009	FIB-SEM	4.2	3.7	109
Shearing [14]	2009	FIB-SEM	10.58	5.39	75
			9.36	4.25	722
Grew [15]	2010	X-ray	22.4	7	2.2
Sumi [7]	2010	FIB-SEM	2.49		1727.6
			2.39		2824
			2.11		2490.6
Shikazono [16]	2010	FIB-SEM	2.556		972
Shearing [17]	2010	FIB-SEM	12.99	8.15	50.5
Guan [18]	2011	X-ray	3.37	2.19	283
Guan [19]	2011	X-ray	4.44		199.439
			3.1		259.2
Wilson [20]	2011	FIB-SEM	3.97		498
			2.84		342
			2.67		530
			2.53		671
Vivet [21]	2011	FIB-SEM	11.2	7.4	957.9
			8.8		349.4
			9.3		634.2
			7.5	4.4	687.1
			11.2	7.4	957.9
			7.2	5.3	703.2
Cronin [8]	2011	FIB-SEM	3.37	2.6	914
			2.5	0.74	1121
Kanno [22]	2011	FIB-SEM	2.11		2424
			1.92		3905
			2.05		17399
Kishimoto [23]	2011	FIB-SEM	2.37		1205
			2.49		1013
			2.44		1664
Chen-Wiegart [24]	2012	X-ray	3.06	2.89	3600
Jiao [25]	2012	FIB-SEM	3.02	2.08	3901.1
			2.48	1.59	3165.9
			2.7	2.36	3608
			2.17	1.81	3428.7
			2.37	2.08	3276.8
			2.12	1.71	2490
Matsui [26]	2012	FIB-SEM	2.49	2.02	1967.3
			1.68	1.28	3369.2
Jiao [27]	2012	FIB-SEM	1.29		3901
			1.02		3165.9
			0.98		2847.6
Kennouche [28]	2013	FIB-SEM	2.98	2.59	5696
			2.09	1.65	5978
			1.58	1.18	5962
			1.21	1.04	7340
			1.88	1.42	5903
			2.21	1.91	6148
Cronin [29]	2013	X-ray	3.06	2.89	3641
Kishimoto [30]	2013	FIB-SEM	1.23	0.345	2495
			1.79	1.53	1754
			1.64	0.297	2203
Kremski [31]	2013	FIB-SEM	3.37	2.89	1000
		FIB-SEM	2.5	1.75	1000
		TXM	1.87	1.45	3000
		TXM	1.17	1.03	3000
Song [32]	2013	X-ray	0.68	0.56	485.514
Viretta [33]	2014	X-ray	4.63	3.07	828.1
			3.46	2.61	46656
Joos [34]	2014	FIB-SEM	2.56		972
		FIB-SEM	4.36		328
		FIB-SEM	1.76		792
		FIB-SEM	2.14		559
Wang [35]	2014	FIB-SEM	6.14	5.16	14.4
Shimura [36]	2014	FIB-SEM	2.47	1.89	2719.8
			2.51	1.65	2197.44
			2.46	1.93	1913.9
			2.15	1.64	2365.4
			2.53	1.64	1713.5
			2.19	1.58	1797.1
			2.61	2.08	1624.9
			3.79	2.83	1964.3
Kubota [37]	2015	FIB-SEM	2.49	2.02	1958.6
			2.12	1.8	666.6
			2.14	1.63	664.9

The TPB densities of the anodes investigated in this study are compared with numerous TPB values for Ni-YSZ materials available from literature [7,8,13–37] (see Figure 3 and Table S1). The TPB densities are plotted *versus* the volume of the analyzed data cube from tomography. It shows that most reported TPB densities are in the range of 1–4 μm^{-2}. The six data points for fine, medium and coarse anodes before and after redox cycling also fit into this "normal" TPB-range (red crosses). The scatter (1–4 μm^{-2}) is mainly attributed to true microstructure variations (*i.e.*, different coarseness of the microstructures and different anode compositions). Some minor scatter may be also due to the fact that different image analysis techniques are used for TPB measurements (*i.e.*, centroid method, volume expansion method, classical edge length). However, Figure 3 also illustrates that in some cases extraordinary high TPB values (>>5 μm^{-2}) are reported. This is only the case, when the analyzed data volume decreases below 1000 μm^3. It is indeed possible that some nanostructured materials do have much higher TPBs, which can be captured with small image windows at high resolution. But for most materials such high TPB-values are not realistic. It is thus probable that the high TPB values are due to the fact that the analyzed data cubes are smaller than the representative elementary volume (REV). In this context, Figure 3 also documents that our own analyses are based on comparably large data cubes (6000–12,000 μm^3).

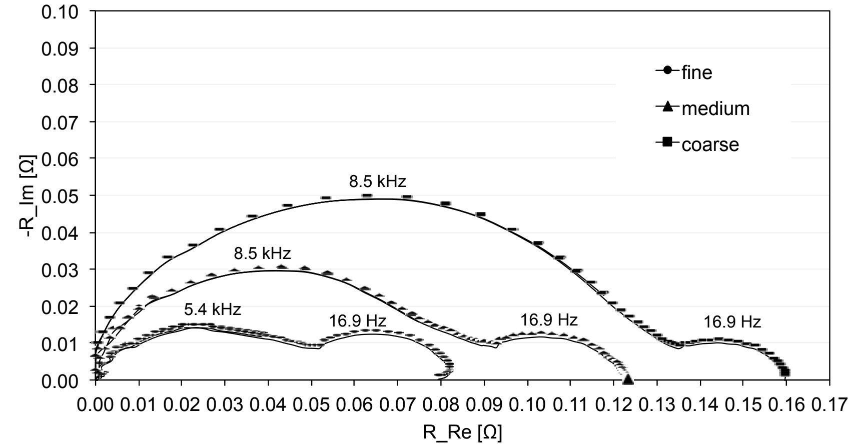

Figure S1. Representative impedance curves for the fine, medium, and coarse samples before redox cycling. The contributions to the polarization resistance are not easy to distinguish by simple inspection of the impedance arcs.

Table S2. An example output from the TPB analysis described in the experimental section.

volume size in	x	y	z	500	500	500
voxel size in	x	y	z	24.41	24.41	25
number for all phases in the image	0	100	200			
number of voxels per phase	45180968	49923115	29895917			
total number of TPB's	927					
total number of TPB voxels	736147					
total length of skeletonized TPB's	3457412					

TPB No.	voxels	length	COG (Center of Gravity) points x	y	z	PHASE 1						PHASE 2						PHASE 3						UNREDUCED VOLUME		REDUCED VOLUME CRITERIA (RVC)			RVC + 111 in all phases	
						x=0	y=0	z=0	x=mx	y=my	z=mz	x=0	y=0	z=0	x=mx	y=my	z=mz	x=0	y=0	z=0	x=mx	y=my	z=mz	VOXEL	LENGTH	x	y	z	VOXEL	LENGTH
1	227	812.2	11887.7	122.1	150	0	1	1	1	0	0	1	1	1	1	1	1	0	1	1	1	0	0	227	812.2	0	0	0	0	0
2	62	144.6	6883.6	73.2	75	1	1	1	1	1	1	1	1	1	1	1	1	1	1	1	1	1	1	62	144.6	1	0	0	0	0
12	28310	135641.4	2245.7	3148.9	3150	1	1	1	1	1	1	1	1	1	1	1	1	1	1	1	1	1	1	28310	135641.4	1	1	1	28310	135641.4
86	577	2742.7	1196.1	4052.1	725	1	1	1	1	1	1	0	0	0	0	0	0	0	0	1	0	0	0	0	0	0	1	0	0	0
:	:	:	:	:	:	:	:	:	:	:	:	:	:	:	:	:	:	:	:	:	:	:	:	:	:	:	:	:	:	:
:	:	:	:	:	:	:	:	:	:	:	:	:	:	:	:	:	:	:	:	:	:	:	:	:	:	:	:	:	:	:
927	1	0	73.2	5297.0	12450	1	0	0	0	0	1	1	1	1	1	1	1	1	1	1	1	1	1	0	0	0	1	0	0	0
TOTAL	736147	3457412																						629394	3018093				477597	2322296
																								TOTAL TPB-active without RVC					TOTAL TPB-active with RVC	

Table S2 shows an example of the output sheet from the TPB analysis. The results include the number of discrete TPB lines, and the corresponding number of voxels and associated length (in metric units). Each TPB line is described by its center of gravity (COG) where the TPB is located in 3D space (in x, y and z coordinates). The connectivity of each phase in each TPB object is checked with respect to the 6 faces of the cube. For our anodes with 3 phases (Ni, YSZ, Pores) multiplied by 6 directions this results in 18 characteristic connectivity checks. This method can also be used to analyze the TPB within a sub-region in the total volume, in order to suppress effects from boundary truncation. Specific criteria (i.e. start and end of the sub-region with the corresponding connectivity criteria in *x*, *y* and *z*-directions) can be introduced. To identify active TPBs in anodes the connectivity

criteria have to consider three specific contacts: with the electrolyte (YSZ), with the current collector (Ni) and with the gas channel (pores). The total TPB length is then evaluated from the total cube volume (without any connectivity check), whereas the active TBP lengths are determined using the specific connectivity check for TPBs in the reduced volume.
